# Climate Change ECHO: Telementoring to Improve Climate Literacy for Health Professionals

**DOI:** 10.1016/j.focus.2022.100051

**Published:** 2022-11-24

**Authors:** Joanna G. Katzman, David Herring, Stefan Wheat, Ralph J. Groves, Briana Kazhe-Dominguez, Chamron Martin, Kent Norsworthy, Jinyang Liu, Sabrina Lord, Laura E. Tomedi

**Affiliations:** 1 Department of Neurosurgery, The University of New Mexico School of Medicine, Albuquerque, New Mexico; 2 Project ECHO, The University of New Mexico, Albuquerque, New Mexico; 3 National Oceanic and Atmospheric Administration, Silver Spring, Maryland; 4 Anschutz Medical Campus, University of Colorado, Aurora, Colorado; 5 Office of Assistant Secretary - Indian Affairs, U.S. Department of the Interior, U.S. Public Health Service, Albuquerque, New Mexico; 6 Presbyterian Health System, Albuquerque, New Mexico; 7 College of Population Health, University of New Mexico, Albuquerque, New Mexico

**Keywords:** Climate change education, ECHO telementoring, climate literacy, health professionals

## Abstract

**Introduction:**

Climate change is a global public health crisis. Most clinicians and public health professionals do not receive adequate education to manage and communicate climate-related health impacts to their patients.

**Methods:**

From July 2021 to February 2022, the Project ECHO Climate Change and Human Health program completed 22 weekly trainings for health professionals. These virtual telementoring sessions were designed to improve both knowledge and self-efficacy about climate-related health impacts and climate change‒related communication skills.

**Results:**

Between July 2021 and February 2022, a total of 804 unique participants (from 44 states and 25 countries) attended the sessions. Participants were nurses (24.7%), physicians (16.8%), and public health professionals (8.5%). They completed weekly Zoom polls measuring their knowledge and self-efficacy. On average, participants strongly agreed or agreed that they had learned skills taught in each session (average percentage of those who strongly agreed or agreed=94.6%, range=66.7%–100.0%). Participants (31%) also completed a weekly postsession survey. A total of 91% rated the sessions as excellent or very good regarding evidenced-basis, and 89% rated sessions as excellent or very good regarding improved communication skills with patients and colleagues.

**Conclusions:**

Given the global climate crisis, the Climate Change and Human Health ECHO is successfully building resources and capacity for clinicians and public health professionals.

## HIGHLIGHTS

• The Climate Change and Human Health ECHO (Extension for Community Healthcare Outcomes) program improves knowledge and self-efficacy for clinicians.
• ECHO telementoring improves climate literacy for health professionals.
• The Climate Change and Human Health ECHO program builds resources and capacity.
• The Project ECHO Climate program fills a large educational training gap in climate education.

## INTRODUCTION

Climate change is a public health crisis affecting millions of people throughout the world and most often affecting the most vulnerable in the population.^1^ There are a plethora of consequential climate-related health impacts, including renal failure and stroke from extreme heat and drought, water- and vector-borne diseases from flooding and temperature variability, and pulmonary and cardiovascular compromise from degraded air quality caused by greenhouse gas pollution and wildfires.^2^^,^^3^ Climate-related eco-anxiety, vicarious trauma (e.g., experiencing extreme weather events such as loss of a home because of wildfire or extreme flooding), and depression comprise many of the mental health consequences of global warming.^2^, ^3^, ^4^

Medical and public health organizations in North America, Europe, and Australia recommend the widespread implementation of a science-based climate curriculum into health professional training programs.^5^, ^6^, ^7^, ^8^, ^9^, ^10^ Although the need for climate-related health education is apparent, available trainings are scarce. Clinicians and public health professionals are generally not well prepared to communicate this emerging, rapidly evolving, and potentially emotionally laden information to their patients.^10^, ^11^, ^12^, ^13^ Recently, many medical and health professional schools are beginning to design evidence-based climate and health education programs.^14^^,^^15^

The weekly Climate Change and Human Health (CCHH) ECHO (Extension for Community Healthcare Outcomes) program began in July 2021 with primary goals to (1) improve knowledge and self-efficacy about climate-related health effects and (2) improve climate change‒related communication skills. This program, also called the CCHH ECHO, began 3 months after the success of an 8-week pilot series held from February to April 2021, which offered preliminary data to suggest that climate change telementoring for health professionals is a beneficial and necessary endeavor.^16^

Project ECHO is a virtual telementoring network whereby health professionals (spokes) develop a community of practice; participate in an all-teach, all-learn environment; and benefit from evidence-based information from an interprofessional (hub) team of subject-matter experts.^17^, ^18^, ^19^

## METHODS

### Curriculum and Weekly Session Format

Given the goals to increase knowledge, self-efficacy, and communication skills, the CCHH ECHO curriculum was developed in concert with experts from the National Oceanic and Atmospheric Administration and the Centers for Disease Control and Prevention^20^^,^^21^ to train clinicians and public health professionals about best practices to prevent and mitigate climate-related health effects and to impart effective climate and health communication skills. Topics included extreme heat, mental health impacts, degraded air quality, and emergency preparedness. This study was reviewed and approved by the University of New Mexico IRB.

Participant outreach and recruitment strategies included collaboration and advertisement with Project ECHO/ECHO Institute and affiliated hubs, the CCHH ECHO website, the Medical Society Consortium on Climate and Health, the National Oceanic and Atmospheric Administration, and the Yale School of Public Health Climate Change and Health Program.^22^, ^23^, ^24^, ^25^, ^26^

Each 60-minute CCHH ECHO telementoring session included a nationally known subject-matter expert who presented a 25–35 minute, evidence-based lecture, followed by a facilitated question-and-answer session. Some sessions included a live simulated case to illustrate the health-related impacts of climate change as well as the importance of effective communication; others included a skills session to train participants regarding available climate mapping and data access tools for educational use with patients.^23^

The digital librarian provided all evidence-based references in the chat during the session. Participants could also access the presentation, the video recording, and an updated Zotero resource library on the CCHH ECHO program webpage within a few days of each session completion.^23^ The ECHO model encourages free use of all materials.^27^

### Registration

Participants registered before the session by entering their demographics, location, and profession. ECHO sessions were delivered via Zoom (Zoom, Inc., San Jose, CA), which tracks participant attendance. Attendance data were deduplicated using e-mail addresses. ECHO staff, presenters, and hub team members were excluded from the analysis.

### Zoom Poll

Zoom polling was used to conduct a real-time evaluation of participant knowledge and self-efficacy pertaining to each session's presentation. Two questions were asked at each session (Table 1).

**Table 1:** Climate Change and Human Health ECHO Curriculum and Zoom Poll Results^a^

Date (m/d/y) and question #	Poll questions	Response rate, %	Strongly agree, *n* (%)	Agree, *n* (%)	Neutral, *n* (%)	Disagree, *n* (%)
The science and tools of climate change						

7/21/21 #1	The “climate change tools” presentation is evidence-based and helps me to visualize effects of climate change.	56.3	57 (67.1)	26 (30.6)	1 (1.2)	1 (1.2)
7/21/21 #2	I learned enough in today's session to feel comfortable to begin using the AirNow tool with patients, and/or colleagues and/or my community.	53.0	19 (23.8)	44 (55.0)	16 (20.0)	1 (1.3)
8/18/21 #1	The “Causes and Effects of Global Warming” presentation provided me with a foundation of knowledge on the drivers and consequences of climate change.	69.8	57 (64.8)	30 (34.1)	1 (1.1)	0 (0.0)
8/18/21 #2	I learned enough in today's session to communicate what is driving climate change and some of the adverse effects of climate change on human health.	66.7	32 (38.1)	43 (51.2)	8 (9.5)	1 (1.2)

Extreme heat						

8/25/21 #1	Today, I learned how climate stressors intersect with historical health inequities and have a better understanding of the concept of climate equity.	65.6	32 (54.2)	25 (42.4)	2 (3.4)	0 (0.0)
8/25/21 #2	Today's presentation helped me understand the concepts of the urban heat island and the effect of the built environment on climate exposure.	64.4	41 (70.7)	16 (27.6)	0 (0.0)	1 (1.7)
9/01/21 #1	The “Climate Change and the Skin” presentation provided me with an understanding of how the distribution and transmission of certain infectious diseases will be impacted by climate change.	67.0	38 (50.7)	37 (49.3)	0 (0.0)	0 (0.0)
9/01/21 #2	Today I learned how climate change may contribute to increased incidence of skin cancers like melanoma.	67.9	49 (64.5)	25 (32.9)	2 (2.6)	0 (0.0)
9/08/21 #1	The “Heat and Human Health: Global Challenges & Local Solutions” presentation gave me an understanding of the relationship between climate change and increasing heat related illness.	60.6	43 (68.3)	18 (28.6)	1 (1.6)	1 (1.6)
9/08/21 #2	Today, I learned how certain populations are more vulnerable to heat stress and about strategies for adapting to heat that may reduce risk.	63.5	50 (75.8)	14 (21.2)	2 (3.0)	0 (0.0)
9/15/21 #1	The “Ecological change and vector-borne disease” lecture provided me with concrete examples of vector borne disease that may be impacted by climate change.	41.3	30 (66.7)	0 (0.0)	1 (2.2)	0 (0.0)
9/15/21 #2	Today's presentation provided me with an understanding of how climate change's impact on ecological factors can result in changing distribution of species and therefore changing vector-borne disease patterns.	53.2	29 (50.0)	26 (44.8)	2 (3.4)	1 (1.7)
9/22/21 #1	Today's case on Extreme Heat increased my knowledge in communicating with my patients and community about the heat-related health effects of climate change.	55.8	29 (54.7)	18 (34.0)	6 (11.3)	0 (0.0)
9/22/21 #2	Today's case on Extreme Heat provided me with some communication skills and tools which I may use in the future to help with my patients and community regarding the heat-related health effects of climate change.	53.7	21 (41.2)	27 (52.9)	3 (5.9)	0 (0.0)

Mental health						

9/29/21 #1	Today, I learned which groups are particularly vulnerable to mental health impacts from climate change as well as strategies for prevention and for improving resilience.	47.9	26 (56.5)	18 (39.1)	2 (4.3)	0 (0.0)
9/29/21 #2	The "Mental Health Impacts of Climate Change" presentation improved my understanding of the ways climate can impact mental health.	49.0	31 (66.0)	16 (34.0)	0 (0.0)	0 (0.0)
10/06/21 #1	The “Eco-Anxiety” presentation gave me a foundation for understanding the concepts of eco-anxiety as one of the mental health related effects of climate change.	55.7	33 (61.1)	18 (33.3)	3 (5.6)	0 (0.0)
10/06/21 #2	Today, I learned how health professionals can care for people experiencing eco-anxiety.	58.8	16 (28.1)	33 (57.9)	6 (10.5)	2 (3.5)
10/13/21 #1	The “Climate Change and Indigenous Mental Health” presentation helped me contextualize how climate change affects the mental health of indigenous populations.	50.6	21 (47.7)	22 (50.0)	1 (2.3)	0 (0.0)
10/13/21 #2	Today, I learned about the concept of climate justice and how it applies to the mental health of populations, such as Indigenous Peoples.	58.6	22 (43.1)	25 (49.0)	4 (7.8)	0 (0.0)
10/20/21 #1	Today's presentation today provided me with information regarding the negative environmental impact that methane production plays in agriculture and farming today.	53.8	24 (49.0)	21 (42.9)	3 (6.1)	1 (2.0)
10/20/21 #2	Today's presentation about the psychological barriers to adapting to a plant-based diet helped me to understand some of the varied ways in which humans rationalize their current lifestyle.	42.9	24 (61.5)	11 (28.2)	3 (7.7)	1 (2.6)

Environmental stewardship and climate resilience						

11/03/21 #1	The “Decarbonizing Health Care” talk helped me understand the interrelationship between health care delivery systems and climate change.	55.1	37 (68.5)	16 (29.6)	1 (1.9)	0 (0.0)
11/03/21 #2	Today, I learned about the different scopes of greenhouse gas emissions and the difference between carbon neutral and net zero.	57.1	23 (41.1)	31 (55.4)	2 (3.6)	0 (0.0)
11/10/21 #1	The “Decarbonizing Health Care Talk Part II” talk helped me to learn about the major activities that healthcare delivery systems are undertaking to reduce Greenhouse Gas (GHG) emissions.	48.9	26 (60.5)	16 (37.2)	0 (0.0)	1 (2.3)
11/10/21 #2	Today, I increased my confidence in what it means to decarbonize the health care sector.	60.2	32 (60.4)	20 (37.7)	1 (1.9)	0 (0.0)
11/17/21 #1	Today's presentation on “Climate Change Through A Resilience Lens” helped me to learn about the concept of ecological resilience.	52.0	31 (79.5)	8 (20.5)	0 (0.0)	0 (0.0)
11/17/21 #2	During today's presentation, I increased my understanding of mitigation, adaptation and transformation as concepts that pertain to climate change resilience.	56.0	29 (69.0)	12 (28.6)	1 (2.4)	0 (0.0)
12/01/21 #1	Today's talk regarding Kaiser Permanente's sustainability efforts provided me with a better understanding of its healthcare sector's climate change mitigation efforts.	44.0%	17 (51.5%)	14 (42.4%)	0 (0.0)	2 (6.1)
12/01/21 #2	During today's talk I learned about many of the campaigns Kaiser Permanente is engaged in regarding decarbonizing their organization.	42.7	16 (50.0)	15 (46.9)	0 (0.0)	1 (3.1)

Degraded air quality						

12/08/21 #1	The “Impacts of Traffic Related Air Pollution” presentation helped me understand the contribution of transportation to air pollution and greenhouse gases, and how this pollution disproportionately impacts communities of color.	64.8	35 (59.3)	20 (33.9)	3 (5.1)	1 (1.7)
12/08/21 #2	The "Carbon Journey" presentation gave me concrete examples of how a healthcare system can tackle its greenhouse gas emissions	54.9	22 (44.0)	26 (52.0)	2 (4.0)	0 (0.0)
12/15/21 #1	Today's talk on “Health Impacts on Air Pollution” increased my knowledge on primary and secondary sources of particulate matter and health.	38.5	23 (76.7)	7 (23.3)	0 (0.0)	0 (0.0)
12/15/21 #2	During today's talk, I learned about ozone formation and how it can negatively affect our health.	38.5	17 (56.7)	13 (43.3)	0 (0.0)	0 (0.0)
1/12/22 #1	Today's lecture increased my understanding of current trends related to degraded air quality and to other health hazards.	82.7	28 (65.1)	13 (30.2)	2 (4.7)	0 (0.0)
1/12/22 #2	I learned that increased levels of ozone and wildfire smoke can lead to harmful effects beyond the lung, including maternal health complications and neurological outcomes.	80.8	31 (73.8)	10 (23.8)	1 (2.4)	0 (0.0)
1/19/22 #1	The “Wildfires, Climate Change and Human Health” presentation helped me to understand the projected future trends between climate change and wildfires.	53.4	19 (48.7)	19 (48.7)	1 (2.6)	0 (0.0)
1/19/22 #2	Today, I learned about the potential health impacts of wildfires.	53.4	25 (64.1)	14 (35.9)	0 (0.0)	0 (0.0)
Emergency preparedness/extreme weather events						

1/26/22 #1	The “Climate and Health - extreme weather events and emergency preparedness” presentation helped me understand the vision and strategy of the CDC's Climate and Health Program	52.9	32 (59.3)	22 (40.7)	0 (0.0)	0 (0.0)
1/26/22 #2	In today's session I learned about the CDC's Building Resilience Against Climate Effects (BRACE) program.	56.9	24 (41.4)	33 (56.9)	1 (1.7)	0 (0.0)
2/02/22 #1	The “Public Health Preparedness in the Context of Climate Change” presentation helped me understand some of the principles of the United States National Response framework.	40.7	20 (36.4)	32 (58.2)	1 (1.8)	0 (0.0)
2/02/22 #2	The lecture today provided me with examples of what I can do to prepare my family and my community for the impacts expected from climate change.	40.7	16 (29.1)	27 (49.1)	11 (20.0)	1 (1.8)
2/09/22 #1	The “Public Health Emergency Preparedness and Maritime Issues Related to Climate Change” presentation helped me understand some of the climate hazards facing the Coast Guard.	45.3	40 (55.6)	30 (41.7)	2 (2.8)	0 (0.0)
2/09/22 #2	The lecture today gave me examples of how the Coast Guard is responding to health threats posed by climate change.	42.8	35 (51.5)	27 (39.7)	5 (7.4)	1 (1.5)

m, month; d, day; y, year; # number; CDC, Centers for Disease Control and Prevention; ECHO, Extension for Community Healthcare Outcomes.

a Data reported are from July 21, 2021 to February 9, 2022.

### Postsession Survey

The program provides a weekly, optional survey to participants, who can also claim no-cost continuing education units embedded within the survey. The survey queries participant satisfaction with the session (*Did the session meet objectives and was the content balanced and evidence-based?* and other questions). Responses from July 21, 2021 to February 9, 2022 surveys were aggregated, and the ECHO evaluation team summarized the number and percentage of specific responses (not unique individuals) (Table 2).

**Table 2:** Climate Change and Human Health ECHO Postsession Average Survey Responses (N=717 Total Responses)

Questions	Poor, *n* (%)	Fair, *n* (%)	Good, *n* (%)	Very good, *n* (%)	Excellent, *n* (%)
How well were the stated objectives met?	0 (0.0)	14 (2.0)	64 (8.9)	226 (31.5)	413 (57.6)
How well did the session deliver balanced and objective, evidence-based content?	2 (0.3)	12 (1.7)	55 (7.7)	204 (28.5)	444 (61.9)
The relevance of the presentation to the session's objective was:	0 (0.0)	12 (1.7)	64 (8.9)	186 (25.9)	455 (63.5)
I intend to apply the knowledge and/or skills I have acquired from this activity to my work when in a team environment.	4 (0.6)	4 (0.6)	88 (12.3)	320 (44.6)	301 (42.0)
I am better able to communicate with other members of a multidisciplinary team because of what I learned in this activity.	4 (0.6)	4 (0.6)	75 (10.5)	341 (47.6)	293 (40.9)

*Note:* Data reported are from July 21, 2021 to February 9, 2022.

ECHO, Extension for Community Healthcare Outcomes.

## RESULTS

### Demographics

During the first 6 months of the CCHH ECHO program, 804 unique participants attended the sessions. The largest percentage of participants (*n*=188, 37.5%) were from New Mexico; however, participants also joined from 44 other U.S. states, Puerto Rico, and the U.S. Virgin Islands. International participants joined from 25 countries. Many participants identified as nurses/nurse practitioners (*n*=198, 24.7%), physicians (*n*=135, 16.8%), or public health professionals (n=68,8.5%). Educators accounted for 7.2% (*n*=58). Of the participants who self-identified their gender, there were 51.6% women, 20.5% men, and <1% gender nonconforming or transgender. The largest percentage of participants identified their race as White (*n*=292, 36.3%), followed by Hispanic, Latino, or Spanish (*n*=83, 10.3%); Asian or Asian Indian (*n*=50, 6.2%); African American (*n*=30, 3.7%); and American Indian/Alaska Native (*n*=29, 3.6%) (Table 3).

**Table 3:** Climate Change and Human Health ECHO Participant Characteristics, July 21, 2021–February 9, 2022 (N=804)

Characteristics	*n* (%)
Gender	
Women	415 (51.6)
Men	165 (20.5)
Nonconforming	3 (0.4)
Transgender	1 (0.1)
Prefer not to answer	8 (1.0)
Missing	212 (26.4)
Race/ethnicity	
White	292 (36.3)
Hispanic, Latino, or Spanish	83 (10.3)
Asian or Asian Indian	50 (6.2)
African American	30 (3.7)
American Indian/Alaska Native	29 (3.6)
Native Hawaiian or other Pacific Islander	3 (0.4)
Other	34 (4.2)
Prefer not to answer	16 (2.0)
Missing	267 (4.0)
Profession	
Nurse/nurse practitioner	198 (24.7)
Physician	135 (16.8)
Public health practitioner	68 (8.5)
Educator	58 (7.2)
Community health worker	39 (5.0)
Behavioral health	31 (3.9)
Physician assistant	5 (0.6)
First responder (paramedic, firefighter, law enforcement)	3 (0.4)
Other	235 (29.2)
Missing	32 (4.0)
Location	
International	194 (24.1)
U.S. states^a^	610 (75.9)
Western states	393 (64.4)
Southern states	102 (16.7)
Northeastern states	84 (13.8)
Midwestern states	31 (5.1)

a Regional includes Western states (Alaska, Hawaii, Washington, Oregon, California, Montana, Idaho, Wyoming, Utah, Colorado, Arizona, and New Mexico), Midwestern states (North Dakota, South Dakota, Kansas, Minnesota, Iowa, Missouri, Wisconsin, Illinois, Indiana, Michigan, and Ohio), Northeastern states (Maine, New Hampshire, Massachusetts, Connecticut, New York, Pennsylvania, and New Jersey), and Southern states (Texas, Tennessee, Oklahoma, Arkansas, Louisiana, Mississippi, Alabama, Maryland, District of Columbia, Virginia, North Carolina, Georgia, and Florida). There was no participation from Nevada, Nebraska, Vermont, Rhode Island, Kentucky, West Virginia, Delaware, and South Carolina. Regional percentages use 610 (the number of U.S. participants) as a denominator. ECHO, Extension for Community Healthcare Outcomes.

### Participant Attendance

Of the 804 unique CCHH ECHO participants, 603 attended once, 115 attended twice, 30 attended 3 times, 47 attended 4 times, and 31 attended 5 times. An average of 11 participants attended 6‒20 times, and 30 participants attended all the 21 sessions.

### Zoom Poll Knowledge and Self-Efficacy Questions

Two real-time Zoom poll questions related to knowledge and self-efficacy were asked during each of the 21 sessions. The average response rate for these questions was 54% of the total participants attending the session. Of these respondents, 55.6% strongly agreed, 39% agreed, and 0.8% disagreed that they achieved the session objectives (Table 1).

### Postsession Surveys

Participants completed the postsession survey 717 times, for a 31% average response rate. Participants were asked whether the program met the stated objectives, was evidence based, and increased their communication skills. A total of 90% rated the sessions as excellent or very good regarding evidence basis, and 89% rated the sessions as excellent or very good regarding improvement of their communication skills with patients and colleagues. More than 75% of participants selected excellent or very good in response to the other questions asked (Table 2).

## DISCUSSION

The CCHH ECHO program has succeeded in improving participant knowledge, self-efficacy, and communication skills while providing interprofessional climate change and health education training to a diverse group of clinicians and public health professionals. For participants who attended regularly, the CCHH ECHO offered a community of practice, a venue for advocacy and coordinated action, and/or an impetus to initiate a climate change‒related program of their own.

Most participants were nurses/nurse practitioners, doctors, and public health professionals, suggesting a real-world need for clinicians and public health professionals to increase their climate knowledge and communication skills.

A unique feature of the CCHH ECHO was the emphasis on acquiring both knowledge and communication skills related to climate change and health literacy. An example of this from the curriculum is the disaster preparedness section, which trained participants regarding increasing extreme weather events such as hurricanes, floods, abrupt freeze conditions, heat waves, and wildfires while including the communication skills necessary to disseminate relevant and actionable information to their patients. If clinicians and other healthcare and public health professionals are able to teach their patients about best practices in disaster preparedness, individuals and communities can begin to mitigate the adverse health effects of future disasters.^28^

Because the ECHO model promotes the diffusion of knowledge and multiplies the dissemination of best practices information, this novel program has proven the potential to benefit many broadly distributed patients and communities.^27^ The authors believe that participants will proceed to educate both their colleagues and patients about the observed and potential future impacts of climate change on human health. Notably, other Project ECHO hubs have already begun to replicate this initiative at Dartmouth-Hitchcock Medical Center, Duke University, and ECHO India.^29^, ^30^, ^31^^.^

### Limitations

Because Project ECHO is a voluntary educational telementoring network open to all healthcare professionals, educators, and climate specialists, the program analysis is open to volunteer bias. Participants who volunteered for the program may be more accepting of the topics covered and may be more likely to use the skills covered. However, the ECHO model can examine the benefits to participants’ knowledge, self-efficacy, practice change, and improvements in communication skills. The CCHH ECHO is very interested in understanding how this method of climate education can increase capacity for knowledge and practice change given the worldwide climate crisis.

It is also important to note that 29% of participants fell in the other professional category. Although we are unable to confirm, it is reasonable to surmise, given the weekly facilitated discussions, that many participants could be community organizers, health policy leaders, legal advisors, and/or communication specialists. Thus, although the predominant groups of participants are nurses and doctors, it is possible that many other essential groups of climate specialists are joining these sessions.

## CONCLUSIONS

Given both the global climate crisis and the identified climate education training gap, this novel CCHH ECHO may continue to be a growing worldwide resource for clinicians, public health professionals, and others in the climate change field. In addition, the CCHH ECHO builds capacity by educating interested learners who can then go on to educate others in this growing field. Future CCHH ECHO studies plan to examine many different aspects of behavior change, including patient education, community outreach, and advocacy.

## Declarations of interest

None.
